# Is Nursing a Profession: Students or Probationers?

**Published:** 1919-03-22

**Authors:** Gladys M. E. Leigh


*To the Editor of* The Hospital.

### IS NURSING A PROFESSION: STUDENTS OR PROBATIONERS?

*To the Editor of* The Hospital.

Sir,—Your correspondent "Ierne," in her courteous reference to my letter, defends her mission as to a probationer's educational status by explaining she had merely postponed the question for future consideration. Surely this is equivalent to reading a book backwards. On two of the points raised by me "Ierne" asks for elaboration. Briefly, I stated that the future career of a probationer may be directed into various channels, therefore practical experience of ways and means is essential. At the onset it is impossible to tell in which direction a nurse's talents and inclinations may lie. Three

### THE EDITOR'S LETTER-BOX—(*continued*).

years' practical work is the minimum training now accepted. Shorten the hours, grant longer leave, but maintain the excellence of the British training-schools by upholding the tradition that no work that leads to efficiency can in any sense be considered derogatory. My third statement, that the long vacations granted to most students are impossible for probationers, will, I think, stand the test of criticism. A nurse's training is essentially practical, the theoretical knowledge required of her is not "in ratio" with that of other professions, consequently she does not devote the same time to theoretical study. When a medical student enters the wards of a hospital, he—or she, as the case may be—ceases to enjoy long vacations. If the nursing staffs are to enter these training-schools on the same footing as medical students, the hospitals will naturally demand proportionate fees. Members of the nursing service, generally speaking, are not drawn from the wealthy classes. Has "Ierne" considered the possible cost to them of her scheme? If nursing is to be reconstructed on this basis, it will first be necessary to secure guarantees that future remuneration will be sufficiently high to justify the increased cost of training. Meanwhile, let every nurse, trained and training, insist upon an eight-hour working day, one day out of seven for rest and recreation, one month's leave in every twelve, and the payment of an adequate salary.

Yours faithfully,

GLADYS M. E. LEIGH.

